# The contribution of dietary and non-dietary factors to socioeconomic inequality in childhood anemia in Ethiopia: a regression-based decomposition analysis

**DOI:** 10.1186/s13104-019-4691-4

**Published:** 2019-10-04

**Authors:** Shimels Hussien Mohammed, Tesfa Dejenie Habtewold, Fatima Muhammad, Ahmad Esmaillzadeh

**Affiliations:** 1 Department of Community Nutrition, School of Nutritional Sciences and Dietetics, Tehran University of Medical Sciences-International Campus, Tehran, Iran; 2 Department of Epidemiology, University of Groningen, Groningen, The Netherlands; 3 Department of Epidemiology and Biostatistics, School of Public Health, Tehran University of Medical Sciences, Tehran, Iran; 4 Obesity and Eating Habits Research Center, Endocrinology and Metabolism Molecular Cellular Sciences Institute, Tehran University of Medical Sciences, Tehran, Iran; 5 Food Security Research Center, Department of Community Nutrition, Isfahan University of Medical Sciences, Isfahan, Iran

**Keywords:** Socioeconomic inequality, Anemia, Ethiopia

## Abstract

**Objective:**

There is a scarcity of evidence on socioeconomic inequalities of childhood anemia in Ethiopia. We determined the magnitude of socioeconomic inequality in anemia and the contribution of dietary and non-dietary factors to the observed inequality, using a nationally representative data of 2902 children included in the 2016 Ethiopian demographic and health survey. The data were collected following a multistage, stratified cluster sampling strategy. We followed the Blinder–Oaxaca regression-based approach to decompose the inequality and determine the relative contribution (%) of the dietary and non-dietary factors to the observed inequality.

**Result:**

We found a significant pro-poor socioeconomic inequality in childhood anemia in Ethiopia. A third (~ 33%) of the inequality was attributable to compositional differences in the dietary determinants of anemia (dietary diversity, meal frequency, and breastfeeding factors). Non-dietary factors like residence place, maternal education, and birth weight) jointly explained ~ 36% of the inequality. Maternal education was the single most important factor, accounting alone for ~ 28% the inequality, followed by rural residence (~ 17%) and dietary diversity (~ 16%). Efforts to narrow socioeconomic gaps and/or designing equity sensitive interventions by prioritizing the poor in health/nutrition interventions stands worth of consideration to reduce the burden of childhood anemia in Ethiopia and beyond.

## Introduction

Anemia persists to be one of the major nutritional problems of children in developing countries. It is a multi-causal problem, with various dietary and non-dietary risk factors [1]. However, the main risk factors of childhood anemia are infection, poor dietary, breastfeeding, and hygiene practices. Poor socioeconomic and education statuses are often the main underlying factors leading to anemia development [1–5]. Besides, socioeconomic inequality has been shown to be linked to various poor health and nutritional outcomes, including anemia. Studies done in developing countries showed a high burden of malnutrition in communities with wide socioeconomic gaps [6, 7]. Understanding the contribution of the drivers of inequality in health/nutritional outcomes could guide the design of equity sensitive interventions [7, 8].

Childhood anemia is a major problem in Ethiopia, with a national prevalence of 57% in 2016 [9]. However, there is a paucity of evidence on socioeconomic inequality of anemia in Ethiopia. Thus, we aimed to determine the degree of socioeconomic inequality in childhood anemia in Ethiopia, using a nationally representative data from the country’s recent demographic and health survey. We also decomposed the inequality and estimated the relative contribution of the main dietary and non-dietary determinants of anemia to the observed inequality.

## Main text

### Methods

#### Data source and study population

We obtained the data from the Ethiopian demographic and health survey (EDHS), conducted in 2016 [9]. EDHS is part of the international demographic and health surveys project, which have been conducted every 5 years in over 90 mid- and low-income countries [10].

#### Study design and sample

The survey, EDHS 2016, was cross-sectional in design and samples were obtained by a two-stage cluster sampling strategy. The primary (first) sampling units were census enumeration areas (EAs). A total of 645 EAs were selected randomly. The secondary sampling units were households. A fixed number of 28 households were randomly selected from each of the selected EAs. All children under-5 years of age found in the selected households were eligible for inclusion in the survey. Following the above procedure, a total of 2902 children aged 6–23 months were included in the survey. More detailed information on the survey methodology is available in the EDHS 2016 final report [9].

#### Outcome variables

##### Anemia

Anemia status was determined by altitude adjusted hemoglobin (Hb) level < 11 g/dL [2].

##### Socioeconomic status

Socioeconomic status was determined by developing a household wealth index following principal component analysis using asset variables [9]. Based on the wealth index, participants were categorized into five socioeconomic groups, with the highest and the lowest quantiles representing the poorest and the wealthiest strata, respectively.

#### Exposure (determinant) variables

The following are the list of the dietary and non-dietary factors used to decompose the observed socioeconomic inequality in anemia:

##### Dietary factors

- Early initiation of breastfeeding: assessed by whether the child received breastfeeding within the first one hour after birth.
- Exclusive breastfeeding: assessed by whether the child received only breast milk during the first 6 months of age.
- Current breastfeeding status (yes, no).
- Dietary diversity: assessed by whether the child’s complementary feeding met the minimum dietary diversity criteria (i.e. diet composed of ≥ 4 food groups) as recommended in the WHO infant and young child feeding guideline [11]. The dietary data were collected by 24 h dietary recall method and further reduced into 7 food groups (namely grains, egg, meat, milk, legumes, vitamin-A rich fruits and vegetables, and other fruits and vegetables).
- Meal frequency: assessed by whether the frequency of the child’s complementary feeding met the minimum meal frequency, which is ≥ 3 times a day for breastfeeding children and ≥ 4 times a day for non-breastfeeding children [9].

##### Non-dietary factors

- Birth size (small, average, large): assessed by the subjective reporting of the mother on the size of the child at the time of birth. Records of exact birth weights are hardly available in developing countries. Thus, in all DHS surveys across the 90 member countries, birth size has been measured as a proxy indicator of birth weight [9].
- Household water supply (improved, unimproved). Pipe water and protected wells were classified as improved water sources. Springs, lakes, ponds, unprotected wells, rivers, and dams were classified as unimproved water sources [9].
- Household toilet facility (improved, unimproved). Ventilated pit latrines and flush toilets were classified as improved facilities. Others facilities like traditional pit latrines were classified as unimproved facilities [9].
- Child sex (male, female).
- Child age (< 12 months, 12–23 months).
- Household size (< 4, 4–8, > 8 individuals).
- Caregiver’s (mother’s) education status (none, primary, secondary^+^).
- Residence place (urban or rural).

#### Statistical analysis

In all analyses, we took into account the complex design of the survey using the Stata *‘SVY’* command; such that, the strata, cluster design and sampling weights of the survey were taken into account in all estimates. Sampling weights were applied to compensate for the unequal probability of sample selection across the sub-national divisions (regions) and ensure the sample resembles the actual population distribution of the country [9]. Adjustment for study design was done to control the effect of the cluster sampling on the variance of the estimates. To examine the relation of the dietary and non-dietary factors with anemia status, bi-variable analyses were conducted using Chi-square test of association. Following the Blinder–Oaxaca regression-based decomposition approach, we decomposed the anemia inequality into its dietary and non-dietary determinants, and provided the relative contribution of the determinants to the observed inequality [12, 13]. All analyses were using Stata 16. Statistical significance was determined at P ≤ 0.05.

### Result

We included a total of 2902 children, of whom 51% were boys. Children under 12 months of age constituted 34% of the sample. The remaining (66%) were aged 12–23 months. Most of the study participants (81%) were from rural areas and of low educated mothers (88%). The overall prevalence of anemia (Hb < 11 g/dL) was 73.7%. Table 1 presents the prevalence of anemia by sociodemographic categories and other variables. There was significant variation in anemia prevalence by wealth categories (P < 0.001), with the highest and the lowest prevalence being among the poorest (85%) and the richest (65%) wealth groups, respectively. There were also significant differences (P < 0.001) in the prevalence of anemia by mothers’ educational status, residence place, child age, toilet facility, breastfeeding, and complementary feeding practices.

**Table 1:** Relation of dietary and non-dietary factors with anemia (weighted n = 2902)

Variables	Frequency (%)	Anemia prevalence (95% CI)	*P*-value*
Wealth category			
Poorest	34	85 (83–87)	< 0.001
Poorer	17	72 (68–76)	
Middle	16	68 (68–76)	
Richer	13	69 (64–74)	
Richest	20	65 (61–70)	
Early initiation of breastfeeding			
No	13	73 (68–79)	0.522
Yes	87	73 (71–75)	
Exclusive breastfeeding			
No	42	78 (73–83)	< 0.001
Yes	58	69 (66–72)	
Current breastfeeding			
No	89	75 (73–78)	0.609
Yes	11	74 (69–78)	
Dietary diversity			
No	85	75 (74–77)	< 0.001
Yes	15	65 (61–70)	
Meal frequency			
No	56	73 (71–76)	0.012
Yes	44	66 (62–70)	
Child sex			
Boy	49	75 (73–78)	0.059
Girl	51	73 (70–75)	
Child age			
< 12 months	34	78 (75–81)	< 0.001
12–23 months	66	72 (70–74)	
Birth size			
Small	28	80 (78–83)	< 0.001
Average	43	71 (69–74)	
Large	29	72 (69–75)	
Residence place			
Urban	19	67 (63–71)	< 0.001
Rural	81	76 (74–77)	
Caregivers education status			
Illiterate	60	76 (74–79)	< 0.001
Primary	28	74 (70–77)	
Secondary^+^	12	63 (57–68)	
Water source			
Not improved	43	71 (68–75)	0.106
Improved	57	74 (71–77)	
Toilet facility			
Not improved	83	75 (73–77)	0.005
Improved	17	69 (65–73)	
Household size			
< 4	14	76 (71–80)	0.109
4–8	74	73 (71–75)	
> 8	12	78 (74–83)	
Residence place			
Urban	19	67 (63–71)	< 0.001
Rural	81	76 (74–77)	

*CI* confidence interval

*P-value: based on Chi-square test of association

The results of the multi-variable regression-based decomposition analysis of the socioeconomic inequality of anemia into its dietary and non-dietary predictors are shown in Table 2. There was a 20 percentage points difference in anemia prevalence between the lowest and highest wealth quantiles. Of the overall socioeconomic inequality between the richest and the poorest wealth groups, ~ 59% was due to differences in compositional factors, i.e. due to variations in the dietary and non-dietary factors between the wealth groups. A third (~ 33%) of the inequality was explained by the dietary determinants (dietary diversity, meal frequency, and breastfeeding factors). The non-dietary factors (like residence place, maternal education, and birth weight) jointly explained ~ 36% of the inequality. Maternal education was the single most important factor, accounting alone for ~ 28% the inequality, followed by rural residence (~ 17%) and dietary diversity (~ 16%).

**Table 2:** Contribution of dietary and non-dietary factors to anemia prevalence between the richest and the poorest

Variable	Difference due to characteristics (E)			Difference due to coefficients (C)		
	Coefficient	%^a^	*P*-value	Coefficient	%	*P*-value
Dietary factors						
Early initiation of breastfeeding	0.002	0.99	0.516	0.212	104.96	0.046
Exclusive breastfeeding	− 0.024	− 11.88	0.009	0.016	7.92	0.009
Current breastfeeding	− 0.011	− 5.50	0.003	− 0.104	− 51.48	0.003
Dietary diversity	0.033	15.84	0.027	− 0.003	− 1.49	0.893
Meal frequency	0.014	6.98	0.041	0.04	19.80	0.303
Non-dietary factors						
Water source	0.004	1.98	0.647	0.088	43.56	0.019
Toilet facility	0.005	2.48	0.858	0.003	1.49	0.822
Birth size	0.005	2.48	0.039	− 0.073	− 36.14	0.021
Mother education	0.057	27.72	0.007	0.012	5.94	0.725
Residence place	0.034	16.83	0.016	− 0.148	− 73.27	0.017
Constant				0.04	19.80	< 0.001
Total	0.119	58.91	< 0.001	0.083	41.09	0.003

^a^Percent out of the total gap between the richest and poorest wealth groups

### Discussion

Our finding showed that there was a significant socioeconomic inequality in anemia among infants and young children in Ethiopia. Specifically, we found a pro-poor type of inequality, such that the prevalence of anemia was more concentrated among the poor than the better-off. Previous studies done in developing have also shown that the burden of anemia and malnutrition in general was high among communities and individuals of low socioeconomic status. Children of low socioeconomic households are more likely to suffer poor health and nutritional outcomes like stunting, anemia, wasting, and nutrient deficiency disorders [7, 14, 15]. Our finding of a high anemia prevalence (73.7%) was consistent with the reports of other studies done in Ethiopia and other developing countries, too [1, 3, 4, 8, 9]. Previous studies done in similar setups showed the presence of a significant socioeconomic inequality not only in anemia but also in other forms of malnutrition like stunting [7, 8, 14]. In another work, we had also shown that there was a significant socioeconomic inequality in childhood stunting in Ethiopia [16]. Studies done in Iran [17], Bangladesh [14, 18], and Kenya [18] also reported pro-poor socioeconomic inequalities in various forms of malnutrition among under-5 children. We found maternal education to be contributing the greatest proportion of the inequality in anemia between the rich and the poor. The finding was consistent with previous studies done in similar settings which also showed as caregivers’ (maternal) education level played a major role in socioeconomic inequality of malnutrition during childhood [16, 17]. Anemia is generally a pro-poor condition all over the world, particularly in developing countries [1, 7]. There are many plausible mechanisms for the association of socioeconomic inequality with a high concentration (burden) of anemia. High socioeconomic inequality is often associated with inequality in health care and its determinants. Where there is socioeconomic inequality, the poor would be less privileged to utilize health-enhancing services, including access and utilization of health care [7, 19]. It could also be easily acknowledged that health literacy, hygiene, breastfeeding and dietary practices would be generally low in poor communities [8, 15].

The findings of this study have important implications for public health policy makings and further studies in Ethiopia and beyond. Narrowing socioeconomic gaps has been identified as one of the key strategies to meet the Sustainability Development Goals by 2030 [20]. The findings of this study might benefit policymakers to know the population groups requiring priority attention, what strategy to pursue and where to allocate public resource. Generally, knowing the magnitude and determinants of socioeconomic inequality in anemia might help to design equity sensitive interventions and contribute to the goal of reducing the burden of anemia. Narrowing socioeconomic gap does not necessarily mean achievement of optimal health and nutritional status [12]. Malnutrition might be highly prevalent in areas with low inequality but poor average economic and health care conditions [12]. Thus, further locally responsive studies are warranted to know which approach (i.e. improving the average health system or narrowing the socioeconomic gap) would be a more feasible and effective one. Meanwhile, pro-poor health and nutrition public investment stands worth of consideration to address the burden of anemia in Ethiopia and other developing countries.

## Limitations

Although anemia is a multi-causal problem, we included only its most common determinants. The study shares the limitations of cross-sectional design; i.e. it precludes making a cause-effect type of relationship. The children’s anemia status was determined by only Hb level, which could not enable to know the specific anemia type the child acquired.

## Data Availability

The data of this work can be accessed on the International DHS program website. Available on: http://dhsprogram.com/data/dataset/Ethiopia_Standard-DHS_2016.cfm.

## Abbreviations

CI: confidence interval
EDHS: Ethiopian demographic health survey
Hb: hemoglobin level
